# A cucurbit[8]uril based supramolecular assembly and its potential applications for the removal of dye and antibiotic from an aqueous medium†

**DOI:** 10.1039/d4ra00347k

**Published:** 2024-03-11

**Authors:** Lulu Shi, Lin Wang, Mingchun Li, Mei Liu

**Affiliations:** a College of Material Science and Engineering, Huaqiao University, Engineering Research Center of Environment-Friendly Functional Materials, Ministry of Education Xiamen 361021 P.R. China mcli@hqu.edu.cn; b School of Chemistry and Life Science, Advanced Institute of Materials Science, Changchun University of Technology Changchun 130012 P.R. China liumei@ccut.edu.cn

## Abstract

CB[8]-based supramolecular assembly, *i.e.*, 2CB[8]·[ZnCl_4_]·4H_2_O (1) (CB[8] = cucurbit[8]uril), was synthesized under solvothermal condition in the presence of [ZnCl_4_]^2−^ anions as a structure inducer. 1 was applied as a high-efficiency absorbent to remove the commonly used dye amaranth (AMR) and an antibacterial drug of broad-spectrum sulfadiazine sodium (SFZ) from the aqueous solution. It showed an excellent removal rate and could remove 96.08% and 96.21% for AMR and SMZ, respectively. The adsorption behaviors were investigated using FT-IR. The differences in IR spectra revealed that the formation of inclusion complexes is the main driving force of adsorption. The phenyl and sulfonyl or sulfone moieties of AMR and SFZ entered the cavity of CB[8] in 1, and the adsorption mechanism could be due to the formation of inclusion complexes of AMR and SFZ in the CB[8] cavities of 1. This work illustrates the application prospects of CB[8]-based supramolecular assembly in environmental protection.

## Abstract 2

A CB[8]-based supramolecular assembly was used as a high efficiency absorbent to remove common use dye amaranth red (AMR) and an antibacterial drug of broad-spectrum sulfadiazine sodium (SFZ) in aqueous solution.

## Graphical abstract

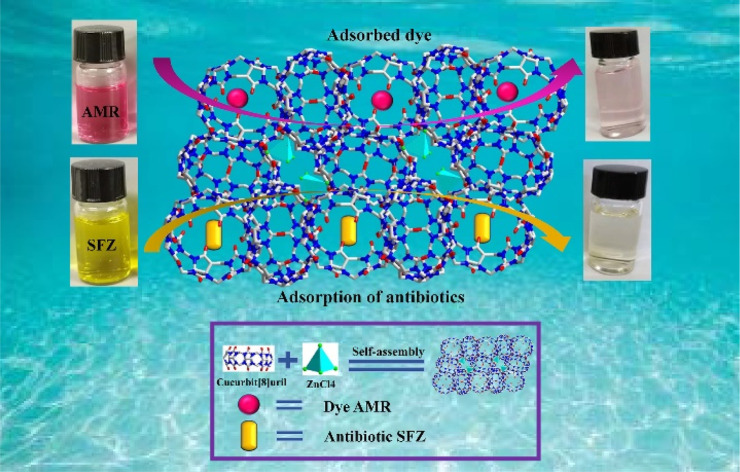

## 1. Introduction

Environmental pollutants have become a serious global environmental problem due to rapid industrialization. Organic dyes, which are widely used in a variety of industries such as textiles, printing, cosmetics, and foodstuffs,^1–4^ have long been recognized as the main source of water pollutants owing to their good water solubility, persistence, biotoxicity and bioaccumulation.^5,6^ Antibiotics, as a treatment for bacterial infections in organisms, are widely used in humans and animals. However, the abuse of antibiotics has caused serious impacts on environmental and human health because of their non-degradability and intrinsic high toxicity. To remove them from aqueous solutions, a variety of technologies, including photocatalysis,^7^ biological degradation,^8^ and physical adsorption,^9^ have been applied. Among them, adsorption is an eco-friendly method for combating dye pollution due to its efficiency and simplicity. Previous research about the adsorbents was mainly focused on activated carbons^10,11^ and zeolites.^12,13^ However, some of these adsorbents suffer from some common issues such as selectivity, reusability, and stability in long-duration operations.

Cucurbit[*n*]urils (CB[*n*]s), as a class organic macrocyclic hosts, can form stable host–guest complexes with various guest molecules such as cationic molecules, ions, and dyes through hydrophobic and electrostatic interaction.^14–16^ Therefore, CB[*n*]s are applicable to remove pollutants from aqueous solutions. In particular, the outer surface of CB[*n*]s with a positive electric potential can interact with other substances, which results in the formation of a variety of CB[*n*]-based supramolecular assemblies.^17–25^ Based on the above background, in this work, we obtained a CB[8]-based supramolecular assembly using ion–dipole interactions between the CB[8] molecules and [ZnCl_4_]^2−^ anions, and the dipole interactions between the adjacent CB[8] molecules. Its capability for the adsorption of dyes and antibiotics was evaluated.

## 2. Experimental

### 2.1. Materials and measurements

CB[8] was synthesized following the literature method.^26^ All other chemicals employed were commercially purchased and used directly without further purification. Thermogravimetric analyses (TGA) were performed in an N_2_ atmosphere with a heating rate of 5 °C min^−1^ with a Netzch STA449F3 analyzer. Powder X-ray diffraction (PXRD) was recorded with a Rigaku/Dmax 2200 pc X-ray diffractometer using Cu-Kα radiation, at a scan speed of 0.2° min^−1^ from 5° to 50°. UV-vis absorption spectra were collected on a Shimadzu UV-2600 spectrophotometer. Fourier-transform infrared (FT-IR) spectra were recorded on a Bruker IFS 66v/S FT-IR spectrometer in the 4000–400 cm^−1^ region.

### 2.2. Synthesis of 2CB[8]·[ZnCl_4_]·4H_2_O(1)

A mixture of CB[8] (0.055 mmol) and ZnCl_2_ (0.4 mmol) was prepared in 4 ml HCl (5 M) and 0.5 ml *N*,*N*-dimethylformamide (DMF), which was heated to 120 °C and kept constant for 2 days in a 10 ml Teflon-lined stainless steel autoclave. Upon slowly cooling the autoclave to room temperature, colourless crystals were obtained.

### 2.3. X-ray crystallography

Single-crystal X-ray data of 1 was collected on a Bruker APX-II CCD area-detector diffractometer with a graphite-monochromated Mo Kα radiation (*λ* = 0.71073 Å) at 210 K. Data reduction was performed using SAINT program and corrected for Lorentz and polarization effects. Adsorption correction was performed *via* the SADABS program.^27^ The structure was solved by direct methods using the program SHELXS-2016. Non-hydrogen atoms were anisotropically refined on *F*^2^ by the full-matrix least-squares technique with SHELXL-2016, and hydrogen atoms were located at geometrically calculated positions.^28^ The highly disordered free solvent molecules in the unit cell were subtracted from the diffraction data with the SQUEEZE subroutine of the PLATON program.^29^ These data were deposited with the Cambridge Crystallographic Data Centre as a supplementary publication No. CCDC 2025014.

## 3. Results and discussion

**Fig. 1:**
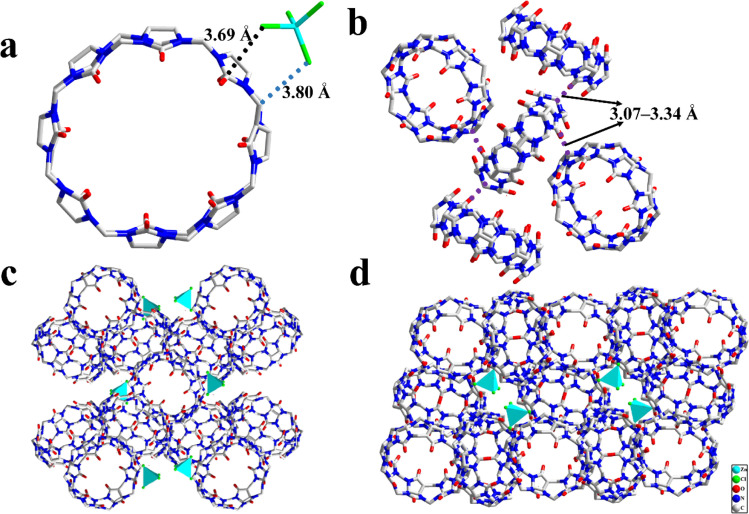
(a) Interactions between [ZnCl_4_]^2−^ complex and CB[8]; (b) interaction between the adjacent CB[8]s; and (c and d) 3D supramolecular structures of 1.

Single crystal X-ray diffraction analysis revealed that 1 crystallizes in a tetragonal system with the space group *I*4_1_/*a* (Table S1†). The formation of such supramolecular assembly is attributed to the ion–dipole interactions between the positive electropotential outer surface of the CB[8] molecules and [ZnCl_4_]^2−^ anions (Fig. 1a), and the dipole interaction between the methylene groups on the outer surface of CB[8]s and the portal carbonyl oxygen of the CB[8]s (Fig. 1b). Thus, the combination of these noncovalent interactions resulted in the formation of the CB[8]-based 3D supramolecular assembly (Fig. 1c and d).

### 3.1. Stability of 1

**Fig. 2:**
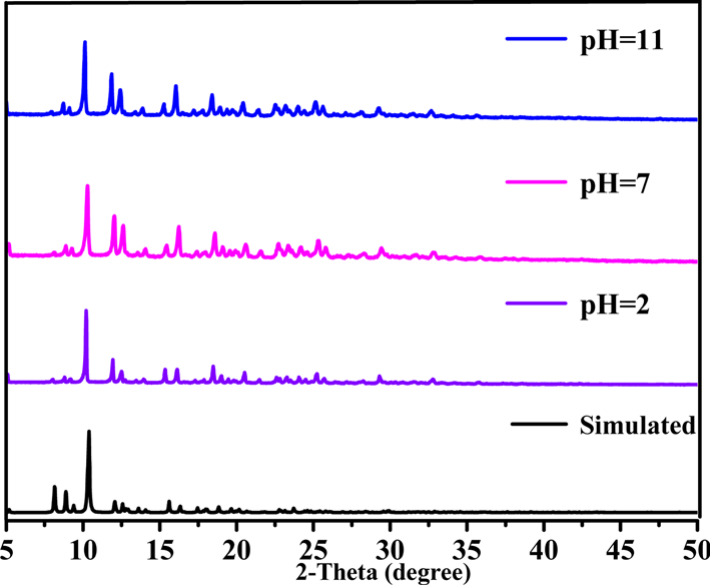
PXRD patterns of 1 after exposure in aqueous solutions with different pH.

The PXRD pattern of the as-synthesized sample is consistent with the simulated one, implying the pure phases of 1. Moreover, to investigate the chemical stability of 1 in water and acidic or alkaline aqueous systems, a 3 mg powder sample of 1 was soaked in aqueous solutions with different pH values for 10 days at room temperature. As presented in Fig. 2, by comparing the PXRD patterns of the simulated 1 after exposure in aqueous solutions with different pH, we found that 1 did not collapse after a long soaking performed, which substantiated that 1 has outstanding chemical stability with a pH value range varying from 2 to 11, which is a crucial prerequisite for the feasible applications as functional materials.

### 3.2. Dye and antibiotic absorption

**Fig. 3:**
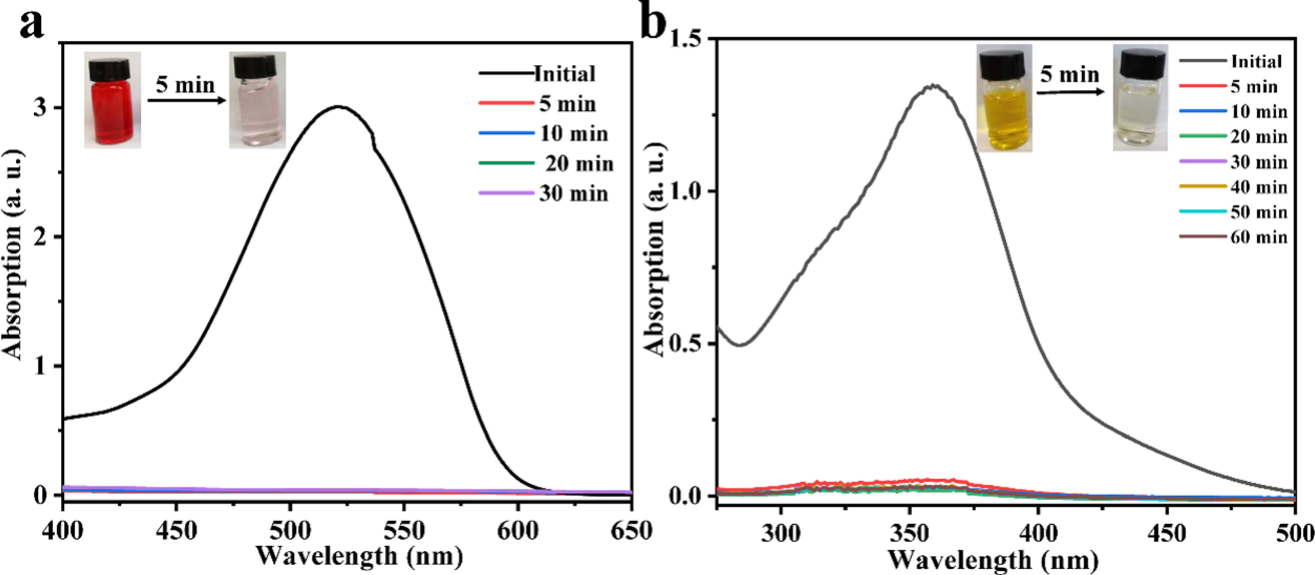
UV/vis absorption spectra of aqueous solution containing AMR (20 mg L^−1^) (a) and SMZ (20 mg L^−1^) (b); insets are images of AMR and SMZ solutions before (left) and after (right) 1 (30 mg) adsorption.

**Fig. 4:**
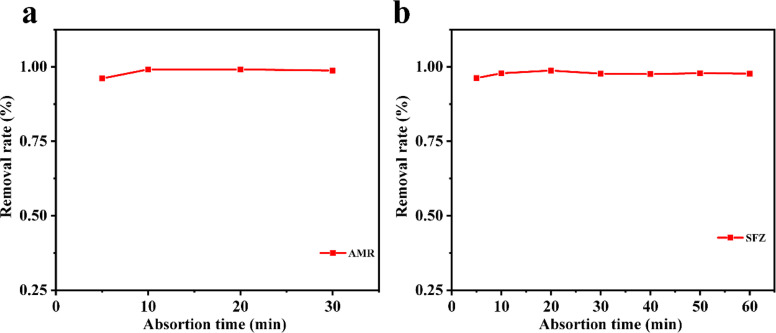
Influence of the adsorption time on AMR (20 mg L^−1^) (a) and SMZ (20 mg L^−1^) and (b) removal with 1 (30 mg).

Considering the big hydrophobic cavity of CB[8] in 1, we investigated the absorption properties of 1 for a common-use dye amaranth (AMR) and an antibacterial drug of broad-spectrum sulfadiazine sodium (SMZ) antibiotic in an aqueous suspension at room temperature. As observed in Fig. 3, the UV/vis absorption spectroscopy revealed that the concentration of AMR and SFZ decreases with time. The removal rate *R* (in%) was calculated according to the eqn:
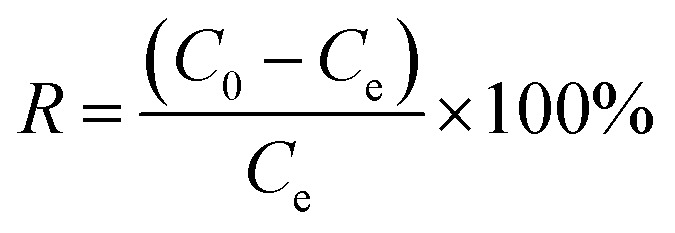
where *C*_0_ and *C*_e_ are the initial and equilibrium concentrations of AMR or SMZ in aqueous solution (in mg L^−1^), respectively. As shown in Fig. 4, the removal rates for AMR and SMZ are as high as 96.08% and 96.21%, respectively. In addition, the aqueous solution of AMR and SMZ became completely colorless in 10 min, indicating the excellent adsorption ability and rapid kinetics of 1 for the removal of AMR and AMZ.

### 3.3. The recyclability of 1

**Fig. 5:**
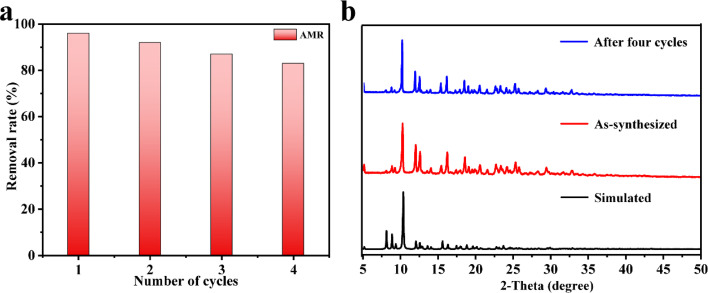
(a) The recyclability tests of 1 towards the adsorption of aqueous AMR (20 mg L^−1^) in water and (b) PXRD patterns of 1 as-synthesized and after four cycles.

Because recyclability is an important performance for adsorbents in practical applications, the recycling experiments were performed using AMR as an example. The sample of 1 was soaked in 5 ml hydrochloric acid solution with pH between 1 and 2 for 1 h to release the absorbed dye. Subsequently, 1 was collected by centrifugation and washed with water several times. The removal rate of 1 after four cycles was basically retained (Fig. 5a, the slight decrease in the removal rates was due to a lower mass loss of 1 during the recycling experiment), indicating that 1 could be reused as an adsorbent to remove AMR for a considerably long time. Moreover, the PXRD pattern of the regenerated 1 was almost the same as the as-synthesized one (Fig. 5b), confirming that the framework of 1 remained stable.

### 3.4. Anti-interference performance of 1

**Fig. 6:**
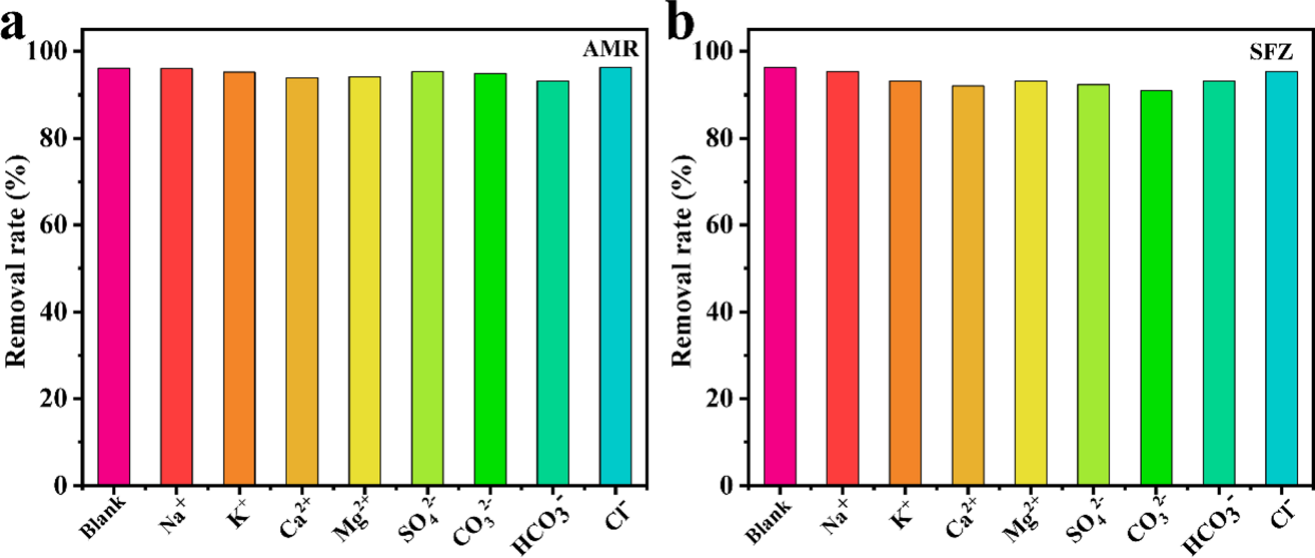
Anti-interference experiment in the presence of other metal ions for AMR (a) and SFZ (b) adsorption (1 × 10^−4^ M^−1^).

The influence of common ions such as K^+^, Ca^2+^, Mg^2+^, Na^+^, SO_4_^2−^, CO_3_^2−^, HCO_3_^−^, and Cl^−^ towards 1 was studied. As shown in Fig. 6, compared with the strong adsorption effect of 1 in ultrapure water for AMR and SFZ, the addition of these ions only caused negligible changes in the removal rates, proving that 1 can be considered a highly efficient adsorbent to remove AMR and SFZ from wastewater.

### 3.5. Adsorption mechanism

**Fig. 7:**
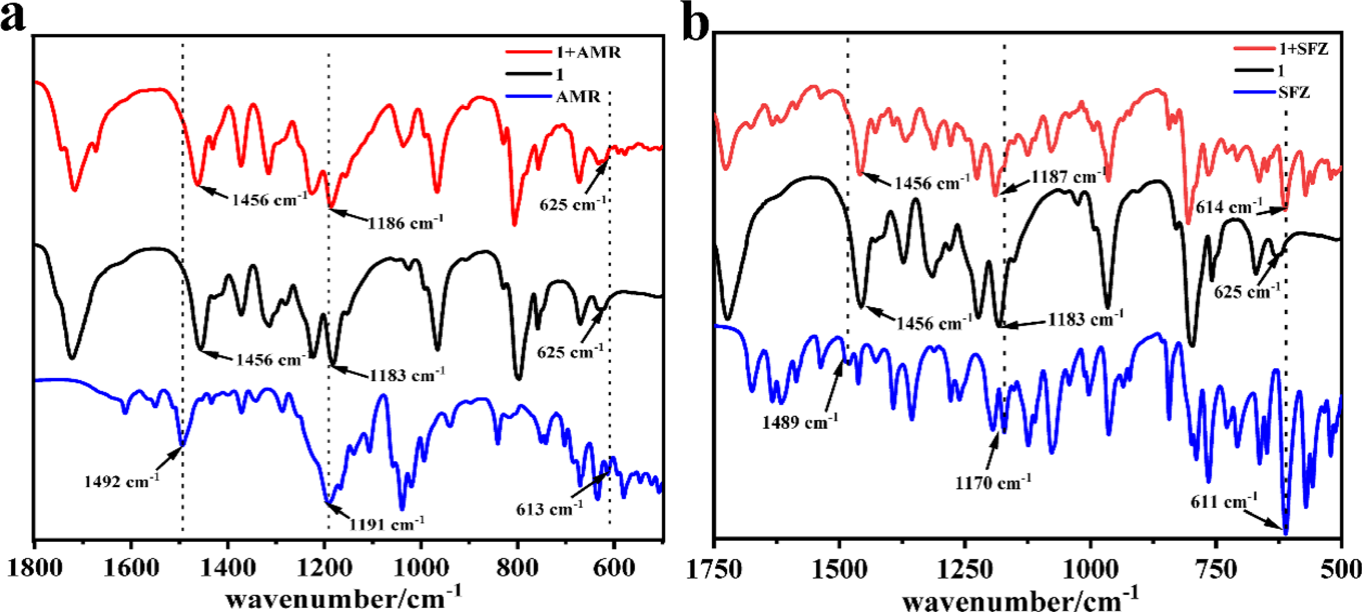
The FT-IR spectra of 1, AMR and 1 + AMR (a) and the FT-IR spectra of 1, SFZ and 1 + SFZ (b).

**Fig. 8:**
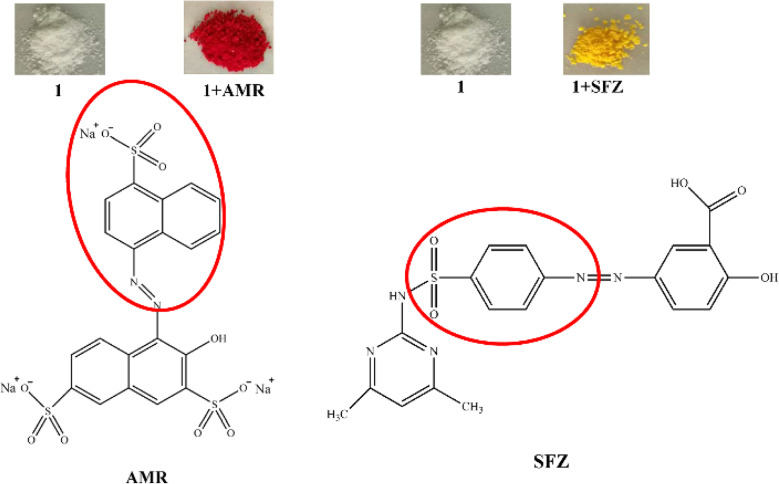
(Top) Photographs of the crystals of 1 before and after AMR and SFZ adsorption, and (bottom) the structure of AMR and SFZ, and their inclusion parts by 1 (the red circle).

The adsorption mechanism was explored by FT-IR (Fig. 7). It can be found that nearly all the obvious absorption bands of 1 and some strong absorption bands of AMR can be observed in 1 + AMR. However, careful observation revealed that the C=C double bond stretching vibration of the phenyl ring appeared at 1494 cm^−1^, the out-of-plane deformations of the benzene ring appeared at 613 cm^−1^, and the asymmetric stretching vibration of sulfonyl at 1191 cm^−1^ of AMR disappeared in 1 + AMR. It may be due to that the phenyl and sulfonate of AMR entered into the cavity of CB[8] in 1, resulting in the disappearance of those bands (Fig. 8a).^30–32^ The same analysis was performed on the FT-IR of 1 + SFZ. The main adsorption bands of 1 and SFZ are still presented in 1 + AMR, while the C=C stretching vibration of phenyl at 1489 cm^−1^ and the S=O stretching vibration of the sulfone moiety at 1489 cm^−1^ disappeared in 1 + SFZ, while the out-of-plane deformations of the benzene ring at 611 cm^−1^ shifted to 614 cm^−1^. These results revealed that the phenyl and sulfone resided inside the cavity of CB[8] in 1 (Fig. 8b).^30–32^

### 3.6. Practical application

**Fig. 9:**
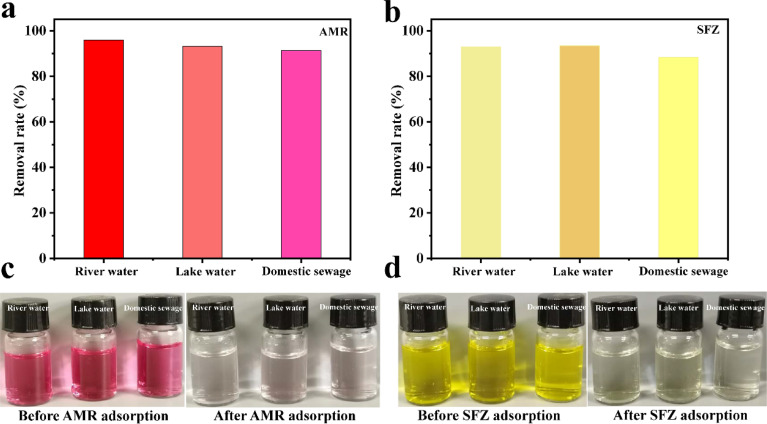
The removal rate of AMR (a) and SFZ (b) in diverse water. The photos of AMR (c) and SFZ (d) solution before adsorption and their supernatant after the adsorption.

To evaluate whether the adsorption of AMR and SFZ is applicable in real samples, the real water sample from the river (Nanhu Park, Changchun, China) was analyzed through the spiked methods. The real water sample from the river water (Duishan community discharge trench, Xiamen, China), lake water (Egret Lake, Xiamen Campus of Huaqiao University, China) and the domestic sewage (Duishan community sewage pipe network, Xiamen, China) were used. The removal rates of AMR and SFZ are 95.82% and 92.82% for river water, 93.16% and 93.37% for lake water, and 91.26% and 88.29% for domestic sewage, respectively (Fig. 9). These practical application results indicate that the analytical method is reliable.

## 4. Conclusions

In summary, a new supramolecular self-assembly based on CB[8] was constructed by a solvothermal method. It showed excellent adsorption performance for AMR and SFZ in the aqueous solution. Remarkably, 1 can adsorb AMR and SFZ with fast speed and reach adsorption saturation within 5 min. The adsorption mechanism was explored using FT-IR spectroscopy, and the investigation revealed that the adsorption mechanism might be due to a new inclusion complex being produced between CB[8] in 1 and AMR or SFZ during the process of adsorption. This study might open up new CB[*n*]-based assemblies in the fields of adsorption materials. Further investigations in this domain are ongoing in our laboratory.

## Conflicts of interest

The authors declare that they have no conflicts of interest.

## Supplementary Material

RA-014-D4RA00347K-s001

RA-014-D4RA00347K-s002
