# Supplementary material for: A cucurbit[8]uril based supramolecular assembly and its potential applications for the removal of dye and antibiotic from an aqueous medium
Source: RSC Adv. 2024 Mar 11;14(12):8161–6. doi: 10.1039/d4ra00347k (PMC10925957; doi:10.1039/d4ra00347k)
Supplement: RA-014-D4RA00347K-s001 [file RA-014-D4RA00347K-s001.pdf]

Electronic Supplementary Material (ESI) for RSC Advances.
This journal is © The Royal Society of Chemistry 2024

Table S1. Crystal data and structure refinement for **1**

Compound	**1**
Empirical formula	$C_{100}H_{104}Cl_4N_{60}O_{36}Zn$
Formula weight	2929.60
Crystal system	Tetragonal
Space group	$I4_1/a$
a (Å)	28.138(4)
b (Å)	28.138(4)
c (Å)	21.379(4)
α (°)	90
β (°)	90
γ (°)	90
V (Å^3)	16926(5)
Z	4
D_c/(g cm^{-3})	1.150
μ/(mm^{-1})	0.287
F(000)	6040.0
θ range (°)	3.756-55.194
R_{int}	0.1149
Parameters	456
T (K)	210.0
goodness of fit	1.039
Limiting indices	$-35 \leq h \leq 35$
	$-36 \leq k \leq 33$
	$-27 \leq l \leq 27$
R indices [I > 2σ(I)]	R_1=0.0695, wR_2=0.2259
R indices (all data)	R_1=0.1407, wR_2=0.2739
